# RESEARCH TO STRENGTHEN, INNOVATE, AND TRANSFORM AGE-FRIENDLY COMMUNITY PRACTICE

**DOI:** 10.1093/geroni/igad104.1506

**Published:** 2023-12-21

**Authors:** Kathy Black

## Abstract

There is much research being conducted to better understand and advance age-friendly community practice. This symposium presents research from leading age-friendly researchers and practitioners across the United States. Drs. Black and Oh provide an analysis of the nation’s sectoral efforts based on progress reported by the age-friendly communities. Drs. Hernandez and Coyle will describe the research and community engagement that led to the development of an aging equity conceptual framework and examples of how it is being operationalized in the City of Boston. Drs. Greenfield and doctoral student Pope will present on a scoping review of studies in the U.S. and Canada on the range of ways in which the public sector participates in age-friendly community efforts. Drs. Coyle and Oh and doctoral students Gleason and Somerville present on a study that explored factors inhibiting communities from officially joining the age-friendly network. Dr. Perry reports on efforts to elevate the voice of older adults on social justice issues pertaining to aging in place in the domain of housing. Individual abstracts provide further detail on each study’s methods and findings.

